# Cardiac dysfunction induced by weaning from mechanical ventilation: incidence, risk factors, and effects of fluid removal

**DOI:** 10.1186/s13054-016-1533-9

**Published:** 2016-11-12

**Authors:** Jinglun Liu, Feng Shen, Jean-Louis Teboul, Nadia Anguel, Alexandra Beurton, Nadia Bezaz, Christian Richard, Xavier Monnet

**Affiliations:** 1 Université Paris-Sud, Faculté de Médecine, Université Paris-Saclay, Le Kremlin-Bicêtre, France; 2 AP-HP, Service de réanimation médicale, Hôpital de Bicêtre, 78, rue du Général Leclerc, 94 270 Le Kremlin-Bicêtre, France; 3 Inserm UMR_S 999, Hôpital Marie Lannelongue, Le Plessis-Robinson, France; 4 Department of Emergency Medicine and Critical Care Medicine, The First Affiliated Hospital of Chongqing Medical University, Chongqing, China; 5 Department of Critical Care Medicine, Affiliated Hospital of Guizhou Medical University, Guiyang, China

**Keywords:** Weaning, Mechanical ventilation, Pulmonary oedema, Heart-lung interactions, Diuretics, COPD

## Abstract

**Background:**

Weaning-induced pulmonary oedema (WiPO) is a well-recognised cause of failure of weaning from mechanical ventilation, but its incidence and risk factors have not been reliably described. We wanted to determine the incidence and risk factors in a population of critically ill patients. In addition, we wanted to describe the effects of diuretics when they are administered in this context.

**Methods:**

We monitored 283 consecutive spontaneous breathing trials (SBT; T-piece trial) performed in 81 patients. In cases with cardiac output monitoring (*n* = 85, 29 patients), a passive leg raising (PLR) test was performed before SBT. Three experts established the diagnosis of WiPO based on various patient characteristics.

**Results:**

SBT failed in 128 cases (45 % of all SBT). WiPO occurred in 59 % of these failing cases. Compared to patients without WiPO (*n* = 52), patients with at least one WiPO (*n* = 29) had a higher prevalence of chronic obstructive pulmonary disease (COPD) (38 % vs. 12 %, respectively; *p* < 0.01), previous “structural” cardiopathy (dilated and/or hypertrophic and/or hypokinetic cardiopathy and/or significant valvular disease, 9 % vs. 25 %, respectively; *p* < 0.01), obesity (45 % vs. 17 %, respectively; *p* < 0.01), and low left ventricular ejection fraction (55 % vs. 21 %, respectively; *p* = 0.01). At logistic regression, COPD (odds ratio (OR) 8.7, 95 % confidence interval (CI) 2.0–37.3), previous structural cardiopathy (OR 4.5, 95 % CI 1.4–14.1), and obesity (OR 3.6, 95 % CI 1.2–12.6) were independent risk factors for experiencing at least one episode of WiPO. In 16 cases with WiPO and a negative PLR at baseline, treatment including diuretics was started. In 9 of these cases, the PLR remained negative before the following SBT. A new episode of WiPO occurred in 7 of these instances, while the two other were extubated. In 7 other cases, the PLR became positive before the following SBT. WiPO did not occur anymore in 6 of these 7 patients who were extubated, while the remaining one was not.

**Conclusions:**

In our population of critically ill patients, WiPO was responsible for 59 % of weaning failures. COPD, previous “structural” cardiopathy, and, to a lesser extent, obesity were the main risk factors. When a treatment including fluid removal had changed preload-independence to preload-dependence, the following SBT was very likely to succeed.

## Background

Failure of weaning from mechanical ventilation increases the length of mechanical ventilation and length of stay in the intensive care unit and it is associated with poor outcome [1, 2]. Among the causes of weaning failure, weaning-induced pulmonary oedema (WiPO) is related to the transition from a positive pressure to a negative pressure regimen of ventilation, which creates unfavourable loading conditions for the heart (increase in right and left ventricular preload, and increase in right and left ventricular afterload) and, potentially, induces myocardial ischaemia [3–5].

The incidence of WiPO has not been clearly established. The studies that reported it were of small size and/or included specific populations of patients [6–11]. Moreover, the risk factors for WiPO and the incidence of myocardial ischaemia as a cause of WiPO have been reported only in studies of small size [6–13]. Our group previously showed that WiPO is highly associated with preload independence at the time of the spontaneous breathing trial (SBT) [9]. In this regard, the effects of fluid removal as a treatment for WiPO need to be described.

In a population of critically ill patients, we aimed at: 1) describing the incidence of WiPO; 2) describing the characteristics of patients who experienced WiPO; 3) investigating myocardial ischaemia as a cause of WiPO; and 4) describing in detail the effects of fluid removal on WiPO. In particular, we wanted to test whether the risk of WiPO decreases when a treatment including fluid removal fosters a preload-dependence status.

## Methods

### Patients

The study was approved by our institutional review board (Comité pour la protection des personnes Ile-de-France VII). All patients were informed about the study and gave their consent to participate. They were included once withdrawal of mechanical ventilation was decided. The only exclusion criterion was tracheostomy.

### Spontaneous breathing trial

The decision to perform a SBT was taken when the following criteria of readiness to start the weaning process were fulfilled: inspired oxygen fraction below 50 %, positive end-expiratory pressure below 5 cm H_2_O, Ramsay score of 2–3, cough during tracheal suctioning, and absence of vasopressor administration.

In all patients, SBT was performed on a “T-piece” connected to an oxygen source. The maximum duration for an SBT was set at 1 h, except in patients with a neurologic deficit where it could be prolonged. The poor tolerance of SBT was defined as the occurrence of dyspnoea and/or an oxygen desaturation and/or hypercapnia and/or heart rate ≥140 beats per minute and/or systolic arterial pressure ≥180 mmHg [14]. If patients failed the SBT, they were reconnected to the ventilator. SBT was repeated the next day, according to the decision of the clinicians in charge, until extubation or death. If they thought SBT failure was due to WiPO, the clinicians in charge administered diuretics before the next SBT. In some patients, they also decided to administer nitrates during the next WiPO or angiotensin-converting enzyme inhibitors before the next SBT. Administration of these treatments was not standardised but left to the discretion of the clinicians in charge of the patient.

### Recorded variables

Before starting the SBT, we recorded some demographic data. In particular, we looked for past hypertension, chronic obstructive pulmonary disease (COPD), dilated, hypertrophic or hypokinetic cardiopathy, significant valvular disease (aortic or mitral insufficiency of grade ≥2, mild or severe aortic and mitral stenosis), cardiac arrhythmias, and coronary artery disease. “Structural cardiopathy” was defined as dilated and/or hypertrophic and/or hypokinetic cardiopathy and/or significant valvular disease. The diagnosis of COPD was established using the following criteria: chronic and progressive dyspnoea, cough, and sputum production [15]. These symptoms were collected from the patient’s previous medical reports or from the words of the patient or their relatives. The patient’s weight was measured using scales every day, and from this we calculated weight gain from admission. Fluid balance over the last 24 h, duration of mechanical ventilation before SBT, respiratory rate, heart rate, and systemic arterial pressure were also recorded. Before starting SBT we also collected the results of arterial blood gas analysis, electrocardiogram, and biological tests including troponin Ic, haemoglobin, and plasma protein concentrations.

Before starting SBT, an echocardiography (CX50, Philips Healthcare, Andover, CA, USA) examination was performed by two investigators (JL and FS). In particular, this assessed the left ventricular ejection fraction (biplane or monoplane Simpson method), E and A waves of the mitral flow, and E’ wave of the external mitral annulus [16].

Before starting SBT, in cases where a transpulmonary thermodilution device was in place (PiCCO2, Pulsion Medical Systems, Munich, Germany), three successive measurements were performed and averaged [17]. We measured cardiac index, global end-diastolic volume, and extravascular lung water [18]. A passive leg raising (PLR) test was also performed [19]. This has been demonstrated to detect preload dependence [20].

The SBT was then started. Before reconnecting the patient to the ventilator, we recorded the same variables as before the SBT, except troponin Ic which was reassessed 4 h after SBT. On the electrocardiogram (ECG) we looked for inversion of T waves and depression or elevation of the ST segment compared to the previous ECG. If the patients completed the SBT correctly, they were extubated. Patients that were extubated but who needed to be reintubated or to be placed under non-invasive ventilation within the next 48 h were classified as failing the SBT.

### Diagnosis of WiPO

The diagnosis of WiPO was established by three experts (NA, JLT, and XM) on a beam of argument. They particularly took into account failure or success of SBT, increases in haemoglobin and plasma protein concentrations [10], increases in heart rate and/or arterial pressure, increases in the ratio of the E and A waves of the mitral flow, and of the ratio of the E wave of the mitral flow over the E' wave of the mitral annulus. In patients with a PiCCO2 device, they also took into account the changes in transpulmonary thermodilution variables during the SBT [8]. The experts were unaware of past medical history, fluid balance and weight changes, occurrence of myocardial ischaemia on ECG, changes in troponin Ic levels, and changes in blood gas analysis. In addition, since we wanted to test whether the risk of WiPO decreased when a treatment including fluid removal changed preload independence to preload dependence, experts were unaware of the results of the PLR test.

### Statistics

Data are expressed as mean +/- standard deviation or median (interquartile range), as appropriate. Comparisons between before and the end of the SBT were assessed with a paired Student’s *t* test or a Wilcoxon signed rank sum test. Comparisons of variables between patients with and patients without WiPO were assessed by a two-tailed Student *t* test or a Mann–Whitney *U* test.

To investigate risk factors for WiPO, we performed a forward logistic regression where the occurrence of at least one episode of WiPO was the dependent variable and where the explanatory variables were a past medical history of COPD, a past medical history of structural cardiopathy, the presence of obesity, and the Simplified Acute Physiology Score (SAPS) II. For these variables, there was a significant difference (*p* < 0.05) at univariate analysis between patients with at least one episode of WiPO and those without. Among the other variables that were different between patients with at least one episode of WiPO and those without, age was not included in the logistic regression because it was correlated with SAPS II and the “low ejection fraction” criterion was not included because it was significantly associated with “structural cardiopathy”. The adjusted odds ratio (OR) and the 95 % confidence interval (CI)) were calculated for all independent factors associated with the risk of presenting at least one episode of WiPO. A *p* value <0.05 was considered significant. The statistical analysis was performed with the software MedCalc 15.2.2 (MedCalc Software bvba, Mariakerke, Belgium).

## Results

### Issue of SBT

**Fig. 1:**
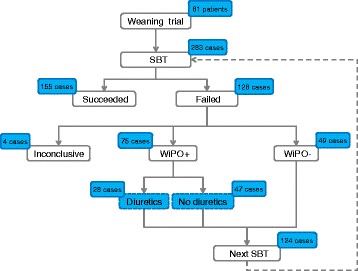
Flow chart. *SBT* spontaneous breathing trial, *WiPO* weaning-induced pulmonary oedema

Eighty-one patients performed 283 SBT. Of these, 155 cases were successful and 128 cases were not (Fig. 1). The planned duration of SBT was 1 h in all cases. In 127 of those 128 deemed to have failed SBT, SBT failed immediately and the patients were not extubated. In one case, SBT seemed to be initially successful and the patient was extubated, but respiratory failure led to reintubation within 48 h. In four cases, the experts could not reach a conclusion regarding the occurrence of WiPO, and they were excluded from further analysis (Fig. 1). WiPO occurred in 75 (27 %) of the SBT, all of which failed the SBT. Thus, WiPO occurred in 59 % of cases with weaning failure. Among the 81 SBT that were performed first in each patient, WiPO occurred in 14 instances. Twenty-nine patients experienced at least one WiPO and 52 patients experienced no WiPO. Among those patients who did not experience any episodes of WiPO, four died and the others were eventually extubated. Patients who experienced at least one episode of WiPO had undergone 6 (interquartile range 3–7) unsuccessful SBT before being extubated, while 2 (interquartile range 1–2) attempts were necessary in patients without any episode of WiPO. Weaning was simple in 35 (43 %) patients, difficult in 19 (23 %) patients, prolonged in 25 (31 %) patients, and 2 (2 %) patients died before being weaned. The incidence of WiPO was 2 %, 32 %, 84 %, and 100 % in these groups, respectively.

### Patient characteristics

**Fig. 2:**
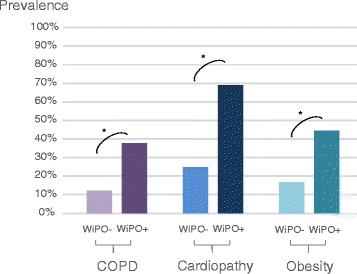
Prevalence of risk factors associated with weaning-induced pulmonary oedema (*WiPO*) depending on the presence (+) or absence (–) of the disease. “Cardiopathy” means dilated and/or hypertrophic and/or hypokinetic cardiopathy and/or significant valvular disease. *COPD* chronic obstructive pulmonary disease. * *p* <0.05 WiPO+ vs. WiPO-

Compared to patients without WiPO, patients with at least one episode of WiPO had a significantly higher prevalence of COPD, “structural” cardiopathy, low left ventricular ejection fraction, and obesity (Table 1, Fig. 2). In two patients without WiPO and four patients with WiPO, obesity had been previously recognised as responsible for a restrictive chronic respiratory failure. At logistic regression, COPD, structural cardiopathy, and obesity were independently associated with the risk of presenting one episode of WiPO (Table 2). SAPS II was not independently associated with the risk of presenting one episode of WiPO (*p* = 0.06).

**Table 1:** Patient characteristics at inclusion

	All patients	Patients with at least one WiPO	Patients without any WiPO	*P* value^a^
	(*n* = 81)	(*n* = 29)	(*n* = 52)	
Age (years)	62 ± 15	67 ± 12	60 ± 16	0.03
Male gender	41 (51 %)	14 (48 %)	27 (52 %)	
Simplified Acute Physiology Score II	47 ± 18	53 ± 22	43 ± 14	0.01
Body mass index (on admission)	27 ± 8	31 ± 10	24 ± 6	<0.01
Obesity	22 (27 %)	13 (45 %)	9 (17 %)	0.01
Aetiology of intubation				
Pneumonia	35 (43 %)	13 (45 %)	22 (42 %)	0.99
Septic shock without pneumonia	27 (33 %)	10 (34 %)	17 (33 %)	0.93
Coma	6 (7 %)	0 (0 %)	6 (7 %)	<0.01
Drug poisoning of other origin	2 (2 %)	0 (0 %)	2 (2 %)	<0.01
Neuromuscular disease	5 (6 %)	0 (0 %)	5 (10 %)	0.07
Cardiogenic shock	4 (5 %)	4 (14 %)	0 (0 %)	0.01
Exacerbation of COPD	2 (2 %)	2 (7 %)	0 (0 %)	0.24
Previous COPD	17 (21 %)	11 (38 %)	6 (11 %)	0.01
Previous cardiovascular diseases				
Hypertension	53 (65 %)	22 (76 %)	31 (60 %)	0.22
Atrial fibrillation	13 (16 %)	9 (31 %)	4 (8 %)	0.01
“Structural” cardiopathy	32 (39 %)	19 (69 %)	13 (25 %)	0.00
Dilated cardiopathy	23 (28 %)	14(48 %)	9 (17 %)	0.00
Hypertrophic cardiopathy	7 (4 %)	4 (3 %)	3 (4 %)	0.41
Significant valvular disease	2 (2 %)	1 (3 %)	1 (2 %)	0.75
Coronary artery disease	13 (16 %)	6 (21 %)	7 (13 %)	0.59
Sepsis-related cardiopathy	6 (6 %)	5 (17 %)	1 (2 %)	0.01
Left ventricular ejection fraction <45 %	27 (33 %)	16 (55 %)	11 (21 %)	<0.01
Total ventilation duration (days)	13 ± 14	20 ± 15	8 ± 12	<0.01
Ventilation duration before the first SBT (days)	6 ± 7	6 ± 6	4 ± 5	0.06
Total duration of ICU stay (days)	17 ± 16	26 ± 16	12 ± 14	<0.01
ICU mortality	6 (7 %)	2 (7 %)	4 (8 %)	0.76

Values are expressed as mean ± standard deviation or number and frequency (%)

^a^Patients with at least one WiPO vs. patients without WiPO

*COPD* chronic obstructive pulmonary disease, *ICU* intensive care unit, *SBT* spontaneous breathing trial, *WiPO* weaning-induced pulmonary oedema

**Table 2:** Summary of the results of forward logistic regression with the occurrence of at least one episode of weaning-induced pulmonary oedema as the dependent variable

Explaining variable	Odds ratio (95 % CI)	*P* value
Previous COPD	8.7 (2.0–37.3)	0.003
Previous structural cardiopathy	4.5 (1.4–14.1)	0.001
Obesity	3.6 (1.2–12.6)	0.03

*CI* confidence interval, *COPD* chronic obstructive pulmonary disease

**Fig. 3:**
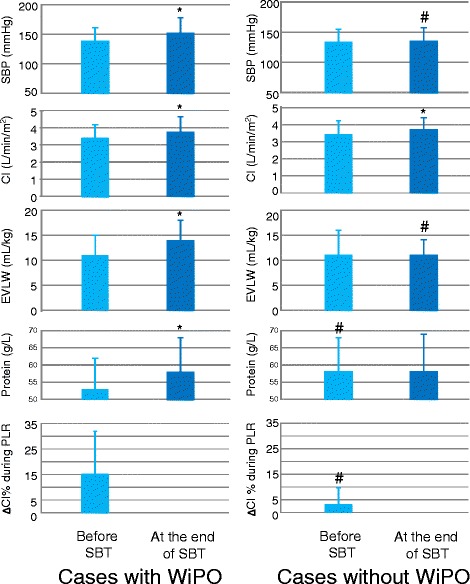
Changes in haemodynamic variables, plasma protein concentration, and extravascular lung water during the spontaneous breathing trial (*SBT*). *p < 0.05 at the end of SBT vs. before SBT; ^#^ *p* < 0.05 cases without WiPO vs. cases with WiPO. *CI* cardiac index, *EVLW* extravascular lung water, *PLR* passive leg raising, *SBP* systolic arterial blood pressure, *WiPO* weaning-induced pulmonary oedema

In 85 of the 283 cases (29 patients), a device for monitoring cardiac output was in place at the time of SBT. Thirty of these cases were accompanied by WiPO. The PLR-induced increase in cardiac index was significantly higher in cases with WiPO than in the others (Table 3, Fig. 3).

**Table 3:** Haemodynamic variables during PLR

				*P* values				
	Before PLR	During PLR	% Change from baseline	During PLR vs. before PLR	Failed SBT vs. succeeded SBT before PLR	Failed SBT vs. succeeded SBT during PLR	Cases without WiPO vs. cases with WiPO before PLR	Cases without WiPO vs. cases with WiPO during PLR
Heart rate (beats/min)								
Succeeded SBT (*n* = 40)	84 ± 17	86 ± 16	4 ± 10 %	0.03				
Failed SBT (*n* = 45)	91 ± 18	94 ± 19	3 ± 10 %	0.06	0.05	0.04		
Cases with WiPO (*n* = 30)	90 ± 16	93 ± 17	4 ± 12 %	0.15				
Cases without WiPO (*n* = 15)	94 ± 22	96 ± 22	3 ± 6 %	0.07			0.49	0.59
Systolic arterial pressure (mmHg)								
Succeeded SBT (*n* = 40)	133 ± 24	141 ± 23	6 ± 8 %	<0.01				
Failed SBT (*n* = 45)	125 ± 20	130 ± 21	5 ± 10 %	<0.01	0.10	0.04		
Cases with WiPO (*n* = 30)	129 ± 18	135 ± 18	6 ± 8 %	<0.01				
Cases without WiPO (*n* = 15)	117 ± 23	120 ± 25	3 ± 12 %	0.35			0.07	0.03
Mean arterial pressure (mmHg)								
Succeeded SBT (*n* = 40)	89 ± 16	95 ± 17	8 ± 10 %	<0.01				
Failed SBT (*n* = 45)	86 ± 12	91 ± 13	6 ± 11 %	<0.01	0.40	0.18		
Cases with WiPO (*n* = 30)	88 ± 12	94 ± 12	7 ± 9 %	<0.01				
Cases without WiPO (*n* = 15)	83 ± 14	85 ± 12	3 ± 14 %	0.53			0.24	0.03
Diastolic arterial pressure (mmHg)								
Succeeded SBT (*n* = 40)	64 ± 12	68 ± 12	7 ± 12 %	0.05				
Failed SBT (*n* = 45)	64 ± 9	69 ± 10	9 ± 12 %	<0.01	0.79	0.85		
Cases with WiPO (*n* = 30)	64 ± 9	71 ± 10	11 ± 11 %	<0.01				
Cases without WiPO (*n* = 15)	63 ± 9	65 ± 9	4 ± 13 %	0.36			0.79	0.09
Cardiac index (L/min/m^2^)								
Succeeded SBT (*n* = 40)	3.4 ± 0.7	3.7 ± 0.7	15 ± 13 %	<0.01				
Failed SBT (*n* = 45)	3.5 ± 0.8	3.8 ± 1.0	7 ± 16 %	0.01	0.24	0.64		
Cases with WiPO (*n* = 30)	3.7 ± 0.9	3.8 ± 0.9	3 ± 7 %	0.06				
Cases without WiPO (*n* = 15)	3.2 ± 0.6	3.7 ± 1.2	17 ± 24 %	0.02			0.03	0.60
Cases with increase in cardiac index ≥10 % during PLR								
Succeeded SBT (*n* = 40)		28 (70 %)						
Failed SBT (*n* = 45)		10 (22 %)				<0.01		
Cases with WiPO (*n* = 30)		2 (7 %)						
Cases without WiPO (*n* = 15)		8 (53 %)						<0.01

Values are expressed as mean ± standard deviation or number and frequency (%)

*PLR* passive leg raising, *SBT* spontaneous breathing trial, *WiPO* weaning-induced pulmonary oedema

### Changes during SBT

The baseline characteristics of all cases with and without WiPO are presented in Table 4. There was no significant difference between SBT with and without WiPO regarding the fluid balance over the last 24 h (Table 4).

**Table 4:** Characteristics of all cases with WiPO and of all cases without WiPO

	Cases with WiPO	Cases without WiPO	*P* value^a^
	(*n* = 75)	(*n* = 204)	
Weight gain since admission (%)	11 ± 15	6 ± 16	0.10
Fluid balance over the last 24 h (mL)	292 (−698 to 1023)	300 (−453 to 1115)	1.00
Mechanical ventilation duration before SBT (days)	11 ± 7	11 ± 10	0.68
Left ventricular ejection fraction at baseline (%)	61 ± 13	57 ± 11	0.76
E/E' ratio of the mitral flow at baseline	10.5 ± 4.3	8.8 ± 3.2*	<0.01
ScvO_2_ at baseline (%)	72 ± 6	72 ± 8	0.86
Duration of SBT (min)	48 ± 17	56 ± 11*	<0.01

Values are expressed as mean ± standard deviation or median (interquartile range)

^a^Cases with at least one WiPO vs. cases without WiPO

SBT spontaneous breathing trial, ScvO_2_ oxygen saturation of the central venous blood, WiPO weaning-induced pulmonary oedema

The duration of SBT was 60 ± 0 min in cases with weaning success, 50 ± 16 min in cases with weaning failure without WiPO (significantly lower than for cases without weaning success), and 48 ± 17 min in cases with weaning failure and WiPO (significantly lower than for cases with weaning success but not different from cases with weaning failure without WiPO). Haemodynamic and biological changes are presented in Tables 5 and 6. The concentration of haemoglobin and of plasma protein at baseline was significantly lower in the 75 cases with WiPO than in the 204 cases without (Table 6).

**Table 5:** Haemodynamic variables before and at the end of SBT

			*P* values				
	Baseline	At the end of SBT	At the end of SBT vs. baseline	Succeeded SBT vs. failed SBT at baseline	Succeeded SBT vs. failed SBT at the end of SBT	Cases with WiPO vs. cases without WiPO at baseline	Cases with WiPO vs. cases without WiPO at the end of SBT
Heart rate (beats/min)							
Succeeded SBT (*n* = 155)	91 ± 15	93 ± 16	0.093				
Failed SBT (*n* = 124)	91 ± 15	101 ± 18	0.000	0.80	<0.01		
Cases with WiPO (*n* = 75)	89 ± 15	102 ± 18	0.000				
Cases without WiPO (*n* = 49)	93 ± 14	99 ± 17	0.011			0.10	0.37
Systolic arterial pressure (mmHg)							
Succeeded SBT (*n* = 155)	134 ± 22	136 ± 22	0.214				
Failed SBT (*n* = 124)	135 ± 23	144 ± 26	0.000	0.70	<0.01		
Cases with WiPO (*n* = 75)	138 ± 23	152 ± 26	0.000				
Cases without WiPO (*n* = 49)	130 ± 22	131 ± 22	0.716			0.06	<0.01
Mean arterial pressure (mmHg)							
Succeeded SBT (*n* = 155)	89 ± 13	88 ± 14	0.478				
ailed SBT (*n* = 124)	90 ± 15	94 ± 18	0.002	0.87	<0.01		
Cases with WiPO (*n* = 75)	92 ± 16	100 ± 18	0.000				
Cases without WiPO (*n* = 49)	87 ± 15	87 ± 15	0.983			0.09	<0.01
Diastolic arterial pressure (mmHg)							
Succeeded SBT (*n* = 155)	67 ± 11	68 ± 12	0.764				
Failed SBT (*n* = 124)	66 ± 12	69 ± 14	0.021	0.53	0.35		
Cases with WiPO (*n* = 75)	67 ± 12	72 ± 14	0.006				
Cases without WiPO (*n* = 49)	65 ± 11	65 ± 12				0.30	0.01
Cardiac index (L/min/m^2^)							
Succeeded SBT (*n* = 40)	3.4 ± 0.7	3.9 ± 1	0.000				
Failed SBT (*n* = 45)	3.2 ± 0.7	3.6 ± 1	0.001	0.13	0.13		
Cases with WiPO (*n* = 30)	3.2 ± 0.7	3.8 ± 1	0.000				
Cases without WiPO (*n* = 15)	3.1 ± 0.6	3.1 ± 1	1.000			0.79	0.02
Global end-diatolic volume (mL/m^2^)							
Succeeded SBT (*n* = 40)	825 ± 254	880 ± 177	0.199				
Failed SBT (*n* = 45)	815 ± 225	916 ± 287	0.002	0.88	0.59		
Cases with WiPO (*n* = 30)	849 ± 133	1032 ± 233	0.000				
Cases without WiPO (*n* = 15)	632 ± 166	624 ± 187	0.718			0.01	<0.01
Extravascular lung water (mL/kg)							
Succeeded SBT (*n* = 40)	10 ± 5	11 ± 3	0.874				
Failed SBT (*n* = 45)	11 ± 3	12 ± 4	0.020	0.91	0.09		
Cases with WiPO (*n* = 30)	11 ± 3	14 ± 4	0.030				
Cases without WiPO (*n* = 15)	9 ± 5	10 ± 1	0.296			0.05	0.01

Values are expressed as mean ± standard deviation

*SBT* spontaneous breathing trial, *WiPO* weaning-induced pulmonary oedema

**Table 6:** Biological variables and electrocardiogram before and at the end of SBT

			*P* values				
	Baseline	At the end of SBT (except for troponin Ic, measured at 4 h)	At the end of SBT vs. baseline	Succeeded SBT vs. failed SBT at baseline	Succeeded SBT vs. failed SBT at the end of SBT	Cases with WiPO vs. cases without WiPO at baseline	Cases with WiPO vs. cases without WiPO at the end of SBT
PaCO_2_ (mmHg)							
Succeeded SBT (*n* = 155)	37 ± 7	38 ± 7					
Failed SBT (*n* = 124)	40 ± 8	46 ± 17	<0.01	<0.01	<0.01		
Cases with WiPO (*n* = 75)	41 ± 9	50 ± 18	<0.01				
Cases without WiPO (*n* = 49)	39 ± 7	41 ± 12				0.11	0.01
PaO_2_ (mmHg)							
Succeeded SBT (*n* = 155)	105 ± 27	97 ± 37	0.03				
Failed SBT (*n* = 124)	96 ± 22	72 ± 17	<0.01	<0.01	<0.01		
Cases with WiPO (*n* = 75)	93 ± 17	71 ± 16	<0.01				
Cases without WiPO (*n* = 49)	101 ± 27	74 ± 20	<0.01			0.05	0.52
Haemoglobin (g/dL)							
Succeeded SBT (*n* = 155)	9.9 ± 1.4	10.0 ± 1.5					
Failed SBT (*n* = 124)	9.4 ± 1.4	10.0 ± 1.5	<0.01	<0.01	0.81		
Cases with WiPO (*n* = 75)	9.1 ± 1.2	10.2 ± 1.3	<0.01				
Cases without WiPO (*n* = 49)	9.7 ± 1.5	9.5 ± 1.6				0.04	0.02
Plasma protein concentration (g/L)							
Succeeded SBT (*n* = 155)	58 ± 11	59 ± 11	0.01				
Failed SBT (*n* = 124)	55 ± 9	57 ± 9	<0.01	0.02	0.19		
Cases with WiPO (*n* = 75)	53 ± 9	58 ± 10	<0.01				
Cases without WiPO (*n* = 49)	58 ± 9	57 ± 8				0.00	0.60
Troponin Ic (ng/mL)							
Succeeded SBT (*n* = 155)	0.07 ± 0.13	0.09 ± 0.06	0.08				
Failed SBT (*n* = 124)	0.10 ± 0.08	0.10 ± 0.06	1.00	0.03	0.17		
Cases with WiPO (*n* = 75)	0.11 ± 0.08	0.11 ± 0.08	1.00				
Cases without WiPO (*n* = 49)	0.07 ± 0.10	0.09 ± 0.09	0.30			0.02	0.20
Patients with increase in troponin Ic >0.5 ng/mL							
Succeeded SBT (*n* = 155)	–	0 (0 %)					
Failed SBT (*n* = 124)	–	0 (0 %)					
Cases with WiPO (*n* = 75)	–	0 (0 %)					
Cases without WiPO (*n* = 49)	–	0 (0 %)					
Patients with changes in electrocardiogram							
Succeeded SBT (*n* = 155)	–	5 (3 %)					
Failed SBT (*n* = 124)	–	5 (4 %)					
Cases with WiPO (*n* = 75)	–	4 (5 %)					
Cases without WiPO (*n* = 49)	–	1 (2 %)					

Values are expressed as mean ± standard deviation or number and frequency (%)

PaO_2_ arterial partial pressure in oxygen , PaCO_2_ arterial partial pressure in carbon dioxide, *SBT* spontaneous breathing trial, *WiPO* weaning-induced pulmonary oedema

During SBT, the increase in PaCO_2_ was significantly higher when SBT failed than if it did not. If SBT failed, the increase in PaCO_2_ was significantly higher in cases with WiPO than in cases without WiPO (Table 6).

The haemoglobin concentration increased in cases with WiPO but not in cases without WiPO, regardless of the success or failure of SBT (Table 6). This was also the case for plasma protein concentration (Fig. 3).

In cases with transpulmonary thermodilution monitoring (*n* = 85), extravascular lung water significantly increased in cases with WiPO but not in cases without WiPO, regardless of the success or failure of SBT (Fig. 3).

### Myocardial ischaemia

Troponin Ic increased more than 0.5 ng/mL in none of the cases (Table 6). Changes in the ECG occurred in 5 cases that succeeded and in 5 cases that failed SBT; among those that failed SBT, the incidence of ECG changes was similar in cases with and without WiPO. All the observed changes in ECG consisted of ST depression of less than 1 mm.

### Treatment and preload dependence

**Fig. 4:**
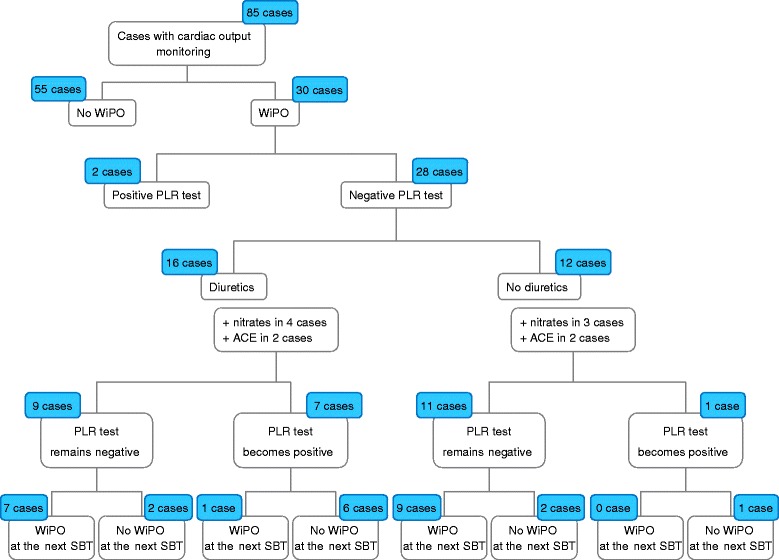
Effects of treatment on the issue of spontaneous breathing trial (*SBT*) depending on the result of a passive leg raising (*PLR*) test. *ACE* angiotensin-converting enzyme, *WiPO* weaning-induced pulmonary oedema

Among the 85 cases (in 29 patients) where a transpulmonary thermodilution device was present, WiPO was seen in 30 of them and did not occur in 55. In 28 of the 30 cases with WiPO, the PLR test was negative. Conversely, in the 55 cases without WiPO, the PLR test was always positive (Fig. 4). Haemodynamic changes during PLR tests are presented in Table 3.

Among the 28 cases with transpulmonary thermodilution where WiPO and a negative PLR test were present, fluid removal by diuretics was started in addition to nitrates and angiotensin-conversting enzyme inhibitor administration in 16 instances (Fig. 4). Among cases where this treatment did not change the result of the PLR test (remaining negative), WiPO occurred again in a very large majority of cases. By contrast, among cases where the PLR test had changed from negative to positive with the treatment, the next SBT succeeded without WiPO in a large majority of cases (Fig. 4).

## Discussion

In our population of critically ill patients, we found that WiPO was responsible for 59 % of cases of weaning failure. Previous COPD, cardiopathy, and, to a lesser extent, obesity were independent risk factors for presenting WiPO. In cases of WiPO, the incidence of myocardial ischaemia during SBT was very low, and it was never accompanied by a significant increase in troponin Ic. After a failed SBT with WiPO, when treatment including fluid removal had changed the PLR test from negative to positive, the following SBT was very likely to succeed.

The studies that investigated WiPO [6–11] did not provide a clear picture of the epidemiology of WiPO and, in particular, how common it is. These studies were limited by the small population size [7, 11] or by the fact that they included only difficult-to-wean patients [7–11]. Moreover, the therapeutic strategy was not systematically investigated in these studies. In this regard, the main advantage of our study is that it included a relatively large population of consecutive patients where the treatment that was undertaken in case of WiPO was clearly described.

In our large series of SBT, WiPO occurred in more than half of all SBT. This result confirms what has been suggested by smaller sized studies [6–11], which reported an incidence of weaning-induced cardiac dysfunction ranging from 44 % [7] to 87 % of weaning failures [6]. This result suggests that WiPO must be recognised as a very common cause of weaning failure. Clinicians should carefully look for this when facing a patient who fails SBT, using one of the several methods that have been described for this purpose. Nevertheless, it is important to take into account that all SBT in our study were performed with a T-piece, a method of SBT that is much more challenging for the heart than pressure support [11].

Previous COPD and “structural” cardiopathy were two factors that were independently and strongly associated with WiPO. Although such conditions are commonly suggested to be associated with WiPO [4, 5, 21], this has rarely been reported [11]. Moreover, as far as we know, our study is the first to demonstrate with logistic regression that these are independent risk factors for WiPO. COPD favours the SBT-induced haemodynamic disturbances that underlie WiPO [4, 21]. Essentially, it is responsible for very large negative swings in intrathoracic pressure during SBT [13], which is one of the *primum movens* of cardiac dysfunction during weaning [4, 5, 21]. Also, the chronic right ventricular failure of these patients may aggravate the deleterious effects of SBT for the right ventricle, such as biventricular interdependence [22, 23].

Obesity was another independent risk factor of WiPO. It was observed in almost half of patients who experienced at least one episode of WiPO and was rarely responsible for previously diagnosed respiratory failure. As far as we know, it has never been reported before. In fact, it seems that body mass index (BMI) was not investigated in previous studies or, at least, not reported. Obesity reduces functional residual capacity and expiratory flows and increases airway resistance [24], all conditions that may contribute to WiPO. However, the association between obesity and the risk of WiPO was weaker than for COPD and previous cardiopathy. This result should ideally be confirmed by future observations.

Another important observation was that myocardial ischaemia (as detected by ECG changes) during SBT was uncommon and that, when it occurred, it was mild, without any increases in troponin Ic. The incidence of myocardial ischaemia as a cause of weaning failure was very different among previous publications [12], ranging from 6 % [25] to 50 % [26]. Many of these previous studies had included a large proportion of patients with coronary artery disease. Although it has been reported that myocardial ischaemia could be the main mechanism of cardiac failure during weaning in some instances, and that its treatment may improve the weaning success in such cases [27], this mechanism is far from common.

Our group previously reported that WiPO is associated with preload independence [9]. To explain this observation, we hypothesised, first, that in case of preload independence of the right ventricle, the increase in venous return and the increase in right ventricular afterload during SBT are likely to result in a further right ventricular dilation. This may lead to a right-to-left shift of the interventricular septum. Second, preload independence of the left ventricle is likely to be associated with left ventricular failure. In this condition, diastolic left ventricular dysfunction is common, so that a further SBT-induced increase in left ventricular preload (volume) may result in a large increase in left ventricular end-diastolic pressure. Furthermore, a failing left ventricle is more sensitive to increases in its afterload. The SBT-induced increase in left ventricular afterload is thus more likely to increase left ventricular end-diastolic pressure.

The present study confirms our previous observation [9]. Furthermore, it describes the effects of fluid removal on the incidence of WiPO, an observation that we did not make in our previous study [9]. In cases of WiPO with a negative PLR, when treatment including fluid removal had changed the PLR test from negative to positive, the success of the next SBT was very likely. In contrast, if the PLR test remained negative, most of the patients failed the next SBT. These results suggest that, when treatment including fluid removal is chosen for treating WiPO, it should be conducted with the goal of reaching a preload-dependent condition. It has been demonstrated that a strategy leading to fluid depletion reduced the duration of ventilation [28]. Nevertheless, definitive proof of the benefit of PLR-guided fluid removal would only come from a study comparing WiPO cases treated with this strategy and other strategies.

A first limitation of our study is that the diagnosis of WiPO was established by the consensual appraisal of experts. However, the experts based their diagnosis on several variables, including some that have been demonstrated to diagnose lung oedema at least as well as the pulmonary artery catheter [9, 10]. As a second limitation, we recorded all the SBT in every patient who underwent more than one SBT. This increased the proportion of unsuccessful SBT. Nevertheless, the analysis of the risk factors for WiPO was based on patients and not on all cases. Moreover, including the same patient for several SBT was necessary to evaluate the therapeutic strategy. Third, the external validity of our single-centre study is limited. In particular, the incidence of weaning failure was high. This is likely explained by the fact that the risk factors for weaning failure in our patients were high. Our population included no post-surgery patients, and more than 75 % of patients had been intubated because of pneumonia or septic shock of another origin. Finally, we did not record the total fluid balance of our patients but used the weight gain as an indirect marker of fluid accumulation.

## Conclusions

In our population of critically ill patients, WiPO was responsible for 59 % of weaning failures. Previous COPD, cardiopathy, and, to a lesser degree, obesity, were independent risk factors for WiPO. These results may warn clinicians about the importance of this diagnosis and should encourage them to look for it in patients at risk. WiPO was associated with a preload-independence condition. When a treatment including fluid removal had changed preload independence to preload dependence, the following SBT was very likely to succeed. It should be further investigated whether, in patients with WiPO, fluid should be removed until preload dependence appears.

## Abbreviations

CI: Confidence interval
COPD: Chronic obstructive pulmonary disease
ECG: Electrocardiogram
OR: Odds ratio
PLR: Passive leg raising
SAPS: Simplified Acute Physiology Score
SBT: Spontaneous breathing trial
WiPO: Weaning-induced pulmonary oedema
