# Erratum to: Cardiac dysfunction induced by weaning from mechanical ventilation: incidence, risk factors, and effects of fluid removal

**DOI:** 10.1186/s13054-017-1634-0

**Published:** 2017-03-08

**Authors:** Jinglun Liu, Feng Shen, Jean-Louis Teboul, Nadia Anguel, Alexandra Beurton, Nadia Bezaz, Christian Richard, Xavier Monnet

**Affiliations:** 1 Université Paris-Sud, Faculté de Médecine, Université Paris-Saclay, Le Kremlin-Bicêtre, France; 2 AP-HP, Service de réanimation médicale, Hôpital de Bicêtre, 78, rue du Général Leclerc, 94 270 Le Kremlin-Bicêtre, France; 3 Inserm UMR_S 999, Hôpital Marie Lannelongue, Le Plessis-Robinson, France; 4 Department of Emergency Medicine and Critical Care Medicine, The First Affiliated Hospital of Chongqing Medical University, Chongqing, China; 5 Department of Critical Care Medicine, Affiliated Hospital of Guizhou Medical University, Guiyang, China

## Erratum

Unfortunately this article was published with an error. During production of the original article [1], “with WiPO” and “without WiPO” on "ΔCI% during PLR" has been switched on Fig. 3. The correct figure is shown below.

**Fig. 3:**
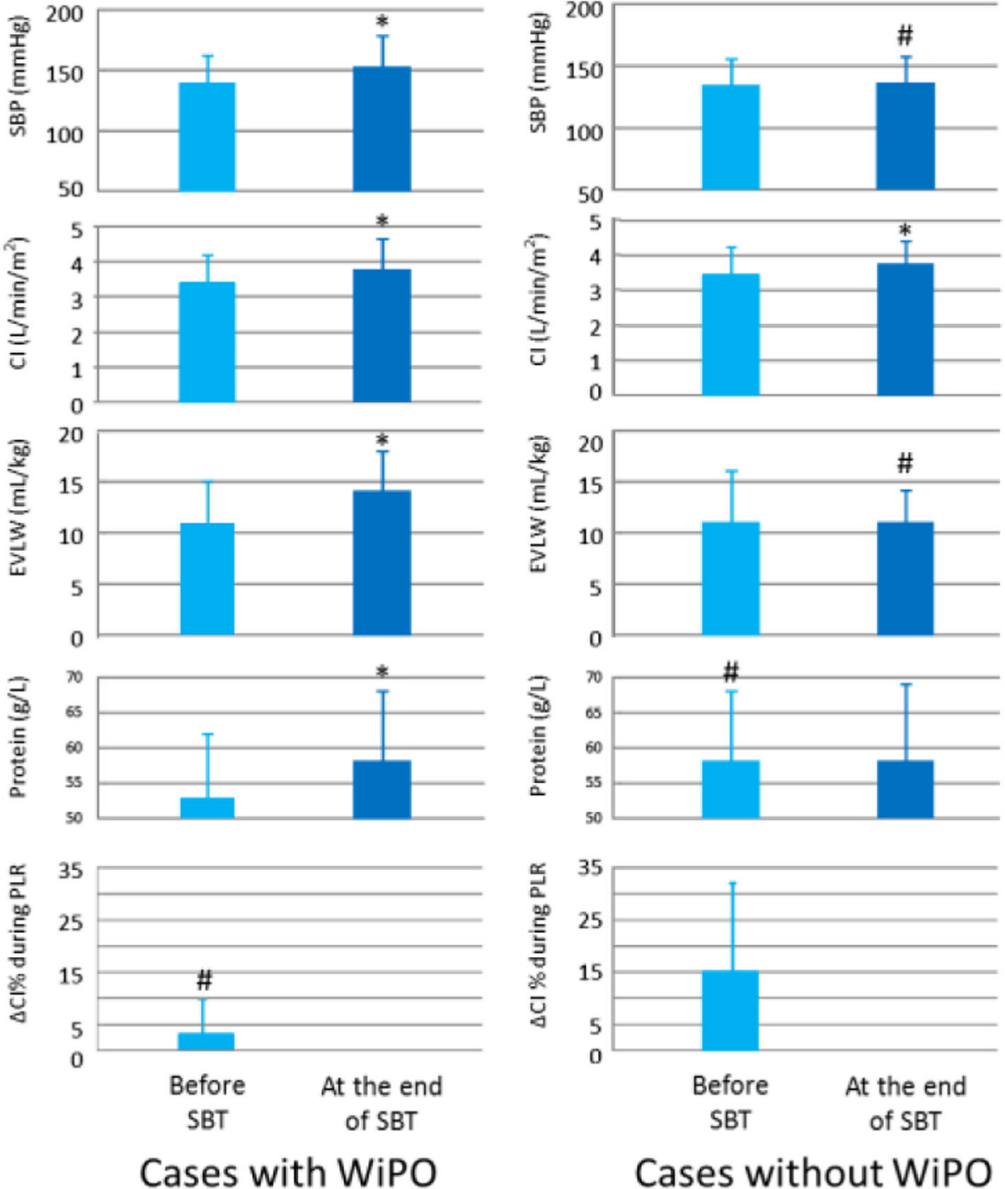
Changes in haemodynamic variables, plasma protein concentration, and extravascular lung water during the spontaneous breathing trial (*SBT*). *p < 0.05 at the end of SBT vs. before SBT; ^#^ *p* < 0.05 cases without WiPO vs. cases with WiPO. *CI* cardiac index, *EVLW* extravascular lung water, *PLR* passive leg raising, *SBP* systolic arterial blood pressure, *WiPO* weaning-induced pulmonary oedema
